# Cognitive therapy as an early treatment for post‐traumatic stress disorder in children and adolescents: a randomized controlled trial addressing preliminary efficacy and mechanisms of action

**DOI:** 10.1111/jcpp.12673

**Published:** 2016-12-15

**Authors:** Richard Meiser‐Stedman, Patrick Smith, Anna McKinnon, Clare Dixon, David Trickey, Anke Ehlers, David M. Clark, Adrian Boyle, Peter Watson, Ian Goodyer, Tim Dalgleish

**Affiliations:** ^1^ Medical Research Council Cognition and Brain Sciences UnitCambridgeUK; ^2^ Department of Clinical PsychologyUniversity of East AngliaNorwichUK; ^3^ Institute of Psychiatry, Psychology and NeuroscienceKing's College LondonLondonUK; ^4^ Anna Freud CentreLondonUK; ^5^ University of OxfordOxfordUK; ^6^ Cambridge University Hospitals NHS Foundation TrustCambridgeUK; ^7^ University of CambridgeCambridgeUK; ^8^ Cambridgeshire and Peterborough NHS Foundation TrustCambridgeUK; ^9^ Present address: Macquarie UniversitySydneyNSWAustralia; ^10^ Present address: University of BathBathUK

**Keywords:** Post‐traumatic stress disorder, cognitive therapy

## Abstract

**Background:**

Few efficacious early treatments for post‐traumatic stress disorder (PTSD) in children and adolescents exist. Previous trials have intervened within the first month post‐trauma and focused on secondary prevention of later post‐traumatic stress; however, considerable natural recovery may still occur up to 6‐months post‐trauma. No trials have addressed the early treatment of established PTSD (i.e. 2‐ to 6‐months post‐trauma).

**Methods:**

Twenty‐nine youth (8–17 years) with PTSD (according to age‐appropriate DSM‐IV or ICD‐10 diagnostic criteria) after a single‐event trauma in the previous 2–6 months were randomly allocated to Cognitive Therapy for PTSD (CT‐PTSD;* n* = 14) or waiting list (WL;* n* = 15) for 10 weeks.

**Results:**

Significantly more participants were free of PTSD after CT‐PTSD (71%) than WL (27%) at posttreatment (intent‐to‐treat, 95% CI for difference .04–.71). CT‐PTSD yielded greater improvement on child‐report questionnaire measures of PTSD, depression and anxiety; clinician‐rated functioning; and parent‐reported outcomes. Recovery after CT‐PTSD was maintained at 6‐ and 12‐month posttreatment. Beneficial effects of CT‐PTSD were mediated through changes in appraisals and safety‐seeking behaviours, as predicted by cognitive models of PTSD. CT‐PTSD was considered acceptable on the basis of low dropout and high treatment credibility and therapist alliance ratings.

**Conclusions:**

This trial provides preliminary support for the efficacy and acceptability of CT‐PTSD as an early treatment for PTSD in youth. Moreover, the trial did not support the extension of ‘watchful waiting’ into the 2‐ to 6‐month post‐trauma window, as significant improvements in the WL arm (particularly in terms of functioning and depression) were not observed. Replication in larger samples is needed, but attention to recruitment issues will be required.

## Introduction

Trauma exposure in childhood and adolescence is common, with post‐traumatic stress disorder (PTSD) occurring in a significant minority (15.9% in a recent meta‐analysis; Alisic et al., 2014). PTSD can take a chronic course in this age group (Yule et al., 2000) and impact significantly on academic and social functioning and mental health in adulthood. Numerous efforts have been made to intervene within the first month post‐trauma to prevent the development of PTSD to single‐event traumas in youth. These have focused on youth attending hospital emergency departments (EDs) and comprised very brief universal interventions (i.e. 1–2 sessions), typically debriefing or psychoeducation, delivered to all trauma‐exposed young people within a month of the trauma. As in adults, these universal approaches demonstrate little or no improvement over no intervention/natural recovery (Marsac, Donlon, & Berkowitz, 2014).

An alternative approach is to intervene early only with those who show initial PTSD symptoms or present with PTSD. Information provision in the first 2 weeks post‐trauma may reduce PTSD symptoms in youth with marked traumatic stress (Kenardy, Cox, & Brown, 2015). In the only meta‐analysis to consider interventions in the first month post‐trauma in youth (Kramer & Landolt, 2011), only one study was found to be efficacious: Berkowitz, Stover, and Marans (2011) evaluated a multi‐session early intervention in youth recently exposed to a trauma, who presented with at least one new PTSD symptom. Their four‐session, cognitive‐behavioural treatment package aimed at improving caregiver‐child communication and trauma‐related coping reduced the likelihood of developing PTSD relative to supportive counselling. Although limited by potential bias (nonblind assessments), inclusion of youth where symptoms may have stemmed from multiple traumas (making onset unclear) and lack of control for the effects of natural recovery, this study suggests that an early, targeted psychological treatment for youth at risk of PTSD is feasible and likely efficacious.

To date, no studies have evaluated treating trauma‐exposed youth with either PTSD or symptoms of PTSD in the early (i.e. 2‐ to 6‐months post‐trauma), but not *acute* (i.e. within a months post‐trauma) period. This early treatment window is important for several reasons. First, it is not known whether treatment in the 2‐ to 6‐months period would have any advantage over natural recovery. One group has found that most recovery occurs by 2–3 months (Le Brocque, Hendrikz, & Kenardy, 2010). However, a recent meta‐analysis suggest that considerable natural recovery can occur up to 6‐months post‐trauma (Hiller et al., 2016). Establishing that *any* active intervention is superior to ‘watchful waiting’ (i.e. a period without intervention where an individual is ‘either encouraged to return for further assessment or offered a specific appointment time’, p18; National Institute for Clinical Excellence, 2005) is an essential first step for the future refinement of early treatment approaches. Second, children and adolescents typically face great barriers in accessing care (e.g. being frequently reliant on parents to refer them to services, a lack of awareness among children themselves that traumatic stress is a recognized mental health difficulty), making treatment in the first month rare in routine settings. Third, PTSD cannot be diagnosed within 4 weeks of a trauma as diagnostic systems recognize that some acute traumatic stress is normal and may recede without intervention. Fourth, adult studies show that treatment may be successful in this early window (i.e. after the first month, but within 6‐months post‐trauma; Ehlers et al., 2003); we, therefore, sought to replicate that finding in children and young people.

In the light of the current lack of data concerning efficacious, brief universal interventions for trauma‐exposed youth, new models for managing early traumatic stress reactions have been proposed. These have included contexts such as large‐scale disasters (McDermott & Cobham, 2014) and accidental injuries seen in emergency departments (Cobham et al., 2012; Kassam‐Adams, 2014). Stepped‐care approaches have, in particular, shown to be efficacious in young children (Salloum et al., 2016). There is also a need for indicated interventions, where treatment is directed towards youth considered to be at high risk of chronic difficulties (Marsac et al., 2014). It is suggested that a study of a multiple‐session intervention for children and adolescents with PTSD in the window described above is an important step in the evolution of the early management of traumatic stress in youth and would support these other efforts. Treatment development would be further accelerated by the examination of mechanisms of action. This study was, therefore, a randomized controlled trial (RCT) that addressed as its primary aim whether Cognitive Therapy for PTSD (CT‐PTSD) is an efficacious early treatment in trauma‐exposed children and adolescents, delivered 2‐ to 6‐months post‐trauma. A CT‐PTSD treatment package tailored for children and adolescents was employed (Smith, Perrin, Yule, & Clark, 2010). CT‐PTSD aims to reverse or ameliorate those mechanisms proposed to maintain PTSD (trauma‐related misappraisals, characteristics of trauma memories, and maladaptive behavioural and cognitive coping strategies; Ehlers & Clark, 2000). This therapy was selected because of its efficacy with youth exposed to single‐event trauma who developed chronic PTSD (Smith et al., 2007), its success as an early treatment for PTSD in adults (Ehlers et al., 2003; from which the youth‐focused CT‐PTSD package was adapted) and its focus on targeting mechanisms found to be involved in the maintenance of PTSD in youth (e.g. Palosaari, Punamaki, Diab, & Qouta, 2013). The control arm in this trial was a waiting list (WL) as (a) there is an absence of any established evidence‐based practice for early treatment with trauma‐exposed youth; and (b) there is a critical need to establish that treatment outperforms natural recovery (a question not addressed by previous trials). Pragmatically, WL also reflects ‘treatment as usual’ for recently exposed youth with PTSD within UK youth mental health services (as treatment would not routinely be offered so soon post‐trauma).

The secondary aim of the trial was to evaluate whether CT‐PTSD led to improvements in comorbid disorders, functioning and parent‐report psychopathology. An exploratory aim was to evaluate whether CT‐PTSD exerts its effects through its proposed mechanisms, i.e. whether effect of treatment was mediated by changes in cognitive mechanisms (as predicted by cognitive models) and/or other more general but plausible psychosocial factors (thereby controlling for possible response bias).

In summary, it was hypothesized that: (a) CT‐PTSD would be superior to WL at posttreatment in terms of PTSD diagnosis (our primary outcome); (b) CT‐PTSD would be superior to WL in terms of self‐reported post‐traumatic stress, depression, and anxiety, clinician‐rated functioning and parent‐reported mental health and behavioural difficulties (our secondary outcomes); and (c) any superiority of CT‐PTSD over WL would be mediated through changes in trauma‐related appraisals, trauma memory characteristics, rumination and safety‐seeking behaviours.

## Method

### Study design

An RCT approved by the UK National Research Ethics Service, Cambridgeshire 1 Research Ethics Committee (10/H0304/11) and registered with the ISRCTN Registry (ISRCTN38352118). Study protocol available from the first author.

### Participants

Participants were recruited from sources across the East of England (covering a broad socioeconomic range, including urban and rural settings), including community mental health teams, family doctors, schools, adverts in health clinics, and emergency departments. Inclusion criteria were (a) 8–17 years old; (b) main presenting problem of PTSD (using an age‐appropriate diagnostic algorithm) relating to a single trauma in previous 2–6 months; and (c) fluency in English. Age‐appropriate diagnoses are commonly used in treatment trials for PTSD in youth (Cohen, Deblinger, Mannarino, & Steer, 2004). Consequently, PTSD was defined, in accordance with evidence‐based practice for 8–18 year olds (Meiser‐Stedman, Smith, Glucksman, Yule, & Dalgleish, 2008; Scheeringa, Wright, Hunt, & Zeanah, 2006), as the presence of one re‐experiencing symptom, one avoidance symptom, two hyperarousal symptoms and impaired functioning. This alternative algorithm (PTSD‐AA) avoided the excessively strict DSM‐IV requirement for three avoidance symptoms that would have applied at the time the trial commenced (since altered for the DSM‐5) but was consistent with the ICD‐10 PTSD diagnosis that was current at the time of trial commencement. As such, all young people in the trial met ICD‐10 PTSD criteria. Pretreatment assessments were conducted by two clinical psychologists (AM and RMS) with extensive experience of assessing trauma‐exposed youth. In addition to confirming the presence or absence of PTSD, these assessments were used to confirm that PTSD was triggered by the recent index event, and not any prior trauma.

Exclusion criteria were as follows: (a) organic brain damage; (b) unconscious >15 min during the trauma; (c) intellectual disability or autistic spectrum disorder; (d) ongoing threat; (e) recently initiated (within 3 months) psychotropic medication; (f) receiving another psychological treatment; (g) acute treatment required for suicide risk or other major mental health problem.

### Procedure

#### Randomization

Participants were randomized to CT‐PTSD or WL. A minimization procedure with stratification according to age (<14 vs. ≥ 14 years), gender, symptom severity (<28 vs. ≥ 28, derived from previous trial; Smith et al., 2007) on the Child PTSD Symptom Scale (CPSS; Foa, Johnson, Feeny, & Treadwell, 2001; Cronbach's α = .89) and pretreatment diagnosis (i.e. meeting both DSM‐IV and PTSD‐AA criteria vs. PTSD‐AA criteria) was used to ensure each trial arm was suitably matched on variables that might moderate outcome.

#### Assessments

Participants were assessed before randomization (‘pretreatment’), 5 weeks postrandomization (‘midtreatment’), 11 weeks postrandomization (‘posttreatment’), and for CT‐PTSD, at 6‐ and 12‐month post‐end‐of‐treatment follow‐up assessments (‘6MFU’ and ‘12MFU’) to see if any treatment gains persisted over time. WL cases were offered CT‐PTSD at the end of the wait period if clinically appropriate and did not, therefore, take part in 6MFU and 12MFU.

Posttreatment interview assessments were administered by postdoctoral‐ and postgraduate‐level psychologists blind to condition; assessors did not deliver treatment or contribute to other elements of the trial and did not work in any of the settings where CT‐PTSD was delivered. Participants were instructed not to disclose their treatment status to the assessor. Blind raters were asked to guess allocation at the end of the interview. For cases where blind raters made a guess (*n* = 23), the agreement with actual allocation was no better than chance (Cohen's *κ *= 09, *p* = .65); this suggests raters were indeed blind. Assessments at 6MFU and 12MFU were not blind as these were only administered to CT‐PTSD participants.

#### Cognitive Therapy for PTSD (CT‐PTSD)

CT‐PTSD was based on a treatment approach (Ehlers et al., 2003) derived from a cognitive model of PTSD (Ehlers & Clark, 2000), with suitable adaptations for youth outlined in a published treatment manual (Smith et al., 2010). Treatment was delivered via up to 10 weekly individual sessions, lasting up to 90 min each. Sessions typically involved the child alone, with parents only joining the session to review what was covered and help to plan homework tasks. Treatment components included the following: psycho‐education, activity scheduling/reclaiming life, imaginal reliving, cognitive restructuring, re‐visiting the site of the trauma, stimulus discrimination with respect to traumatic reminders, direct work with nightmares, image transformation techniques and behavioural experiments. CT‐PTSD places particular emphasis on the close integration of cognitive restructuring with reliving. The manual includes guidance for tailoring treatment to a child's developmental needs (e.g. choice of clinical metaphors, age‐appropriate techniques for undertaking restructuring). The current programme did not include relaxation training or other arousal reduction techniques for treating PTSD symptoms. Therapy sessions were discontinued when, after discussion with the participant and review of session by session CPSS scores, it was agreed that participants had no significant further symptoms to address.

CT‐PTSD was delivered by two clinical psychologists (RMS and AM) who completed a 3‐day training in CT‐PTSD. Both of these therapists had completed a clinical training that was primarily cognitive behavioural in orientation and were no more than 2 years postqualification at the start of the trial; moreover, each had completed doctoral research projects that concerned cognitive processes in childhood PTSD. Fortnightly telephone supervision (by PS) addressed treatment adherence as well as clinical issues and was supplemented by reviewing clinical notes.

### Measures

The Children's PTSD Inventory (CPTSDI; Saigh et al., 2000), a child‐report structured interview, assessed the primary outcome of PTSD‐AA at pretreatment, posttreatment, 6MFU and 12MFU. The CPTSDI has excellent internal consistency, test–retest reliability, and interrater reliability (Saigh et al., 2000). The CPTSDI was also used to derive a continuous measure of PTSD symptomatology (i.e. symptom count, range 0–17; Cronbach's α = .71 for this sample) and an ICD‐10 diagnosis of PTSD. Reliability of posttreatment PTSD‐AA diagnoses was examined using a subset of six randomly selected interviews by a qualified clinical psychologist with extensive experience of assessing trauma‐exposed youth; complete agreement was obtained.

The Anxiety Disorders Interview Schedule for the DSM–IV: Child Version (ADIS‐C; Silverman & Albano, 1996), a structured interview schedule that assesses for emotional and behavioural disorders, was administered at pretreatment to index comorbid diagnoses on the basis of parent and child report (administered separately).

Several secondary outcomes were included. The Children's Global Assessment Scale (CGAS; Shaffer et al., 1983) provided a clinician‐rated measurement of general functioning. Youth‐reported PTSD severity was assessed with the CPSS (Cronbach's α = .87 for this sample), depression with the Mood and Feelings Questionnaire (MFQ; Cronbach's α = .94, Wood, Kroll, Moore, & Harrington, 1995; .94 for this sample), and anxiety was assessed with the Spence Child Anxiety Scale (SCAS; Spence, 1998; Cronbach's α = .92; .93 for this sample). Parents completed the Strengths and Difficulties Questionnaire (SDQ; Goodman, 2001), an index of child emotional difficulties, conduct and hyperactivity (Cronbach's α = .66, .60 and .67, respectively; .81, .63 and .78 for this sample). Youth rated their therapist alliance using the Therapeutic Alliance Scale for Children, revised (TASC‐r; Shirk & Saiz, 1992; subscale Cronbach's α = .67–.74; .74 for total scale in this sample). Youth‐reported treatment credibility was assessed using items adapted from an adult PTSD trial (Ehlers et al., 2003; Cronbach's α = .86 for this sample). Intellectual ability was assessed at pretreatment using two subtests of the Wechsler Abbreviated Scale of Intelligence (Wechsler, 1999).

For mediation analyses, measures of cognitive mechanisms proposed by cognitive models to underlie the maintenance of PTSD were administered at pre‐, mid‐ and posttreatment. The cognitive mechanisms assessed were (a) trauma‐related misappraisals [Child Post‐Traumatic Cognitions Inventory (CPTCI); Meiser‐Stedman et al., 2009; Cronbach's α > .86; .96 for this sample]; (b) trauma memory characteristics [Trauma Memory Quality Questionnaire (TMQQ); Meiser‐Stedman, Smith, Yule, & Dalgleish, 2007; Cronbach's α > .76; .61 for this sample]; and (c) unhelpful coping strategies including rumination (a three‐item scale; Meiser‐Stedman et al., 2014; Cronbach's α = .85; .64 for this sample) and safety‐seeking behaviours (a novel 22‐item measure devised for this trial; Cronbach's α = .96). Measures of generic psychosocial risk factors – social support [Multidimensional Scale of Perceived Social Support (MSPSS); Zimet, Dahlem, Zimet, & Farley, 1988; Cronbach's α = .88; .92 for this sample] and self‐blame (a two‐item scale devised for this study; Cronbach's α = .94) – were also administered.

### Data analysis

Sample size was determined by power calculations based on an estimated recovery rate (loss of diagnosis) of 92% for CT‐PTSD versus 42% for WL derived from our trial of CT‐PTSD versus WL for chronic PTSD (Smith et al., 2007). Assuming these recovery rates, 13 children per group would provide 80% power to detect a difference between CT‐PTSD and WL with α = .05 (two‐tailed).

An intent‐to‐treat (ITT) approach to the analysis of primary and secondary outcomes was adopted, conducted by an independent statistician, with analyses for completers (participants who completed measures at posttreatment) also reported. For categorical (diagnostic) outcome data, including the primary outcome variable (child‐reported PTSD‐AA at posttreatment), chi‐square (or Fisher's exact) tests were performed. In order to account for missing diagnostic data, both ‘last observation carried forward’ (LOCF; assumes missing cases retain their baseline diagnosis) and more conservative ‘worst case scenario’ (WCS; assumes that treatment arm cases do not recover while waiting list cases do recover) procedures are reported.

For ITT analyses with continuous outcomes, a multiple imputation procedure was used to account for data lost through drop out, the value of which we assume relates to observed variables in the data. This procedure used a chained equations multiple imputation procedure (Van Buuren, 2007) and five imputations, conducted in SPSS 22.0 (IBM Corp., Armonk, NY, USA). We incorporated the pretreatment score and allocation group in modelling. Pooled regression estimates and their standard errors were computed across imputations using Rubin's rules (Rubin, 1987). ANCOVAs were carried out on post‐CT‐PTSD/WL data to detect any between‐groups differences at posttreatment, accounting for pretreatment symptom levels.

Between‐groups (CT‐PTSD vs. WL) and within‐subjects (pre–post) effect sizes are reported for primary and secondary outcome measures. Within‐subjects and between‐groups effect sizes were calculated on the basis of pooled standard deviations (Cohen's d). Following Jacobson, Follette, and Revenstorf (1984), clinically significant change was considered to have occurred when a participant's posttreatment CPSS score was more than two standard deviations below the sample baseline score. Indices of clinically significant change were calculated on an ITT basis, with missing values derived using multiple imputation.

Mediation analysis used bootstrap procedures to test the magnitude of any indirect effects. This method compensates for the lack of power associated with small samples by re‐sampling data (Preacher & Hayes, 2008). Only data from trial completers were used as investigations of mechanism require participants to have received an adequate dose of the treatment. Confidence intervals for indirect effects were calculated using 5000 resamples (with replacement). Pretreatment CPSS and mediator scores were accounted for in each analysis. Two mediation strategies were utilized. First, to examine the replicability of the mediation effect shown for CT‐PTSD previously (Smith et al., 2007), the ability of change in putative mediator variables across treatment to mediate the relationship between allocation and change in CPSS scores across treatment was investigated. Second, to adhere to the requirement that the mediator temporally precedes the outcome and reflect a change occurring during treatment (Kraemer, Wilson, Fairburn, & Agras, 2002), the ability of pre–mid treatment changes in putative mediator variables to mediate the relationship between allocation and posttreatment CPSS was examined.

## Results

### Sample and participant flow

**Figure 1:**
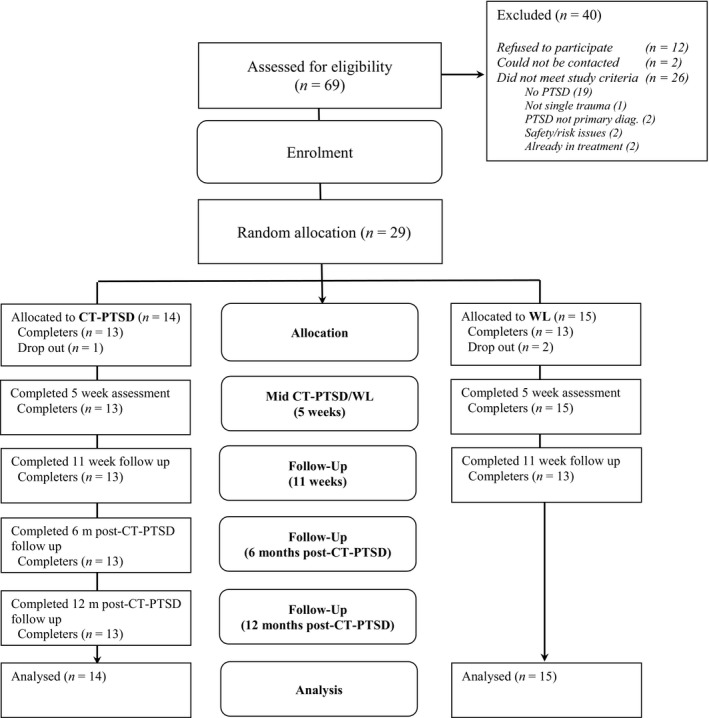
Participant progress (CONSORT flowchart)

Demographic information for *N* = 29 participants who entered the study is presented in Table 1. Figure 1 presents the CONSORT diagram illustrating participant flow. Between 1st April 2011 and 31st August 2013, 132 young people were referred to the study. Of these, 63 were not further assessed after initial telephone screening [sought treatment for another condition (*n* = 18), trauma too long ago (*n* = 16), unable to contact (*n* = 7), other primary diagnosis (*n* = 5), ongoing threat (*n* = 4), too old/young (*n* = 3), multiple trauma (*n* = 3), not interested (*n* = 3), already in treatment (*n* = 3) and ongoing legal action (*n* = 1)]. There were no age or sex differences between youth who were assessed and youth who were excluded at this initial screening (*p* > .55). Of 69 cases assessed for suitability, 29 entered the trial (reasons for nonentry detailed in Figure 1); the majority were community referrals (*n* = 19), the remainder ED attendees who were followed up post‐trauma (*n* = 10). Youth who met inclusion criteria but declined to participate in the trial (*n* = 12) did not differ significantly from trial participants with respect to age, sex or CPSS score (*M* = 24.56, *SD* = 6.98; *p* > .1). Fourteen participants were allocated to CT‐PTSD, 15 to WL. The mean number of therapy sessions in the CT‐PTSD arm was 8.3 (*SD* = 2.2). One CT‐PTSD participant did not complete the midtreatment questionnaires (opting to start medication treatment for depression and to halt PTSD treatment); the same CT‐PTSD participant and two from the WL arm dropped out before completing the posttreatment assessment (one participant did not give a reason for withdrawal; the other felt that trial participation was no longer necessary). No participants allocated to WL reported starting psychological therapy or psychoactive medication.

**Table 1:** Sample description at randomization

	Total sample (*n* = 29)		WL (*n* = 15)		CT‐PTSD (*n* = 14)		Group effect
	Mean/Freq.	*SD*/%	Mean/Freq.	*SD*/%	Mean/Freq.	*SD*/%	
Female	21	72.4	12	80.0	9	64.3	*χ* ^2^ = .90, *p* = .34
Age	13.3	2.5	12.5	2.6	14.2	2.3	*t* = 1.89, *p* = .07
Ethnicity							
White British	25	86.2	11	73.3	14	100.0	*χ* ^2^ = 4.33, *p* < .04
Minority ethnicity	4	13.8	4	26.7	0	0.0	
Household income (per annum)							
<£20,000	12	46.2^a^	9	60.0	3	27.3^a^	*χ* ^2^ = 2.74, *p* = .10
>£20,000	14	53.8^a^	6	40.0	8	72.7^a^	
IQ	96.0	14.7	96.1	17.3	95.9	12.0	*t* = .05, *p* = .96
Any prior trauma	11	37.9	4	26.7	7	50.0	*χ* ^2^ = 1.68, *p* = .20
Prior mental health problems	9	31.0	5	33.3	4	28.6	Fisher's exact test, *p* = .68
Prior mental health treatment	4	13.8	3	20.0	1	7.1	Fisher's exact test, *p* = 1.00
Referral source							
ED screening	10	34.5	4	26.7	6	42.9	*χ* ^2^ = 5.21, *p* = .52
Mental health service	8	27.6	5	33.3	3	21.4	
General practitioner	4	13.8	2	13.3	2	14.3	
Hospital	1	3.4	1	6.7	0	0.0	
Children's services	2	6.9	2	13.3	0	0.0	
School nurse	3	10.3	1	6.7	2	14.3	
Self‐referral	1	3.4	0	0.0	1	7.1	
Trauma type							
Motor vehicle collision	15	51.7	7	46.7	8	57.1	*χ* ^2^ = 4.51, *p* = .48
Assault	7	24.1	3	20.0	4	28.6	
Medical emergency	1	3.4	1	6.7	0	0.0	
House fire	1	3.4	1	6.7	0	0.0	
Other	5	17.2	3	20.0	2	14.3	
Days since trauma	116.7	38.6	125.5	30.3	107.3	45.1	*t* = 1.28, *p* = .21
Ongoing injury at assessment	9	31.0	4	26.7	5	35.7	*χ* ^2^ = .44, *p* = .51
Attended ED	18	62.1	9	60.0	9	64.3	*χ* ^2^ = .06, *p* = .81
Ongoing legal/police issues?	16	55.2	9	60.0	7	50.0	*χ* ^2^ = .29, *p* = .59
PTSD‐AA	29	100.0	15	100.0	14	100.0	n/a
ICD‐10 PTSD	29	100.0	15	100.0	14	100.0	n/a
DSM‐IV PTSD	22	75.9	12	80.0	10	71.4	Fisher's exact test, *p* = .68
Any comorbid disorder							
Anxiety	25	86.2	13	86.7	12	85.7	Fisher's exact test, *p* = 1.00
Affective	16	55.2	7	46.7	9	64.3	Fisher's exact test, *p* = .46
Behavioural	15	51.7	7	46.7	8	57.1	Fisher's exact test, *p* = .72

ED, emergency department; PTSD‐AA, PTSD‐alternative algorithm; IQ, intelligence quotient; WL, waiting list.

a Three missing.

### Pretreatment CT‐PTSD/WL comparisons

At trial entry, the CT‐PTSD and WL groups were matched on all stratification variables (Tables 1 and 2), salient demographic and trauma characteristics and other measures of psychopathology, excepting ethnicity (where CT‐PTSD participants were significantly less likely to belong to a minority ethnic group).

**Table 2:** Outcome measures for completers at each assessment

Outcome variable	WL			CT‐PTSD			Group effect^a^
	*M*	*SD*	*n*	*M*	*SD*	*n*	
PTSD symptoms (CPTSDI)							
Pre	11.7	2.9	15	11.9	3.2	14	*F*_(1,28)_ = .01, *p* = .92
Post	10.2	4.3	13	2.6	3.6	13	*F*_(1,25)_ = 28.90, *p* < .0001
6MFU				1.5	2.6	13	
12MFU				2.1	3.6	13	
PTSD severity (CPSS)							
Pre	30.1	11.5	15	31.1	7.9	14	*F*_(1,28)_ = .08, *p* = .78
Post	24.3	16.0	13	6.2	10.5	13	*F*_(1,25)_ = 16.36, *p* < .0006
6MFU				5.2	8.6	13	
12MFU				5.5	6.2	13	
Depression (MFQ)							
Pre	27.8	16.6	15	29.3	11.6	14	*F*_(1,28)_ = .08, *p* = .78
Post	25.0	21.5	13	11.2	14.0	13	*F*_(1,25)_ = 4.66, *p* < .04
6MFU				11.3	10.9	13	
12MFU				8.2	7.2	13	
Anxiety (SCAS)							
Pre	54.0	25.8	15	45.6	18.4	14	*F*_(1,28)_ = 1.01, *p* = .32
Post	49.5	34.5	13	19.5	18.0	13	*F*_(1,25)_ = 7.50, *p* < .02
6MFU				16.2	15.4	13	
12MFU				14.2	10.1	13	
Functioning (CGAS)							
Pre	58.5	10.5	15	57.3	8.6	14	*F*_(1,28)_ = .12, *p* = .73
Post	55.2	13.8	13	77.4	14.2	13	*F*_(1,25)_ = 17.44, *p* < .0004
Emotional difficulties (SDQ)							
Pre	6.3	3.3	15	5.8	2.4	13	*F*_(1,27)_ = .23, *p* = .64
Post	5.6	3.5	11	3.3	2.1	13	*F*_(1,22)_ = 8.53, *p* < .01
Conduct problems (SDQ)							
Pre	4.2	2.3	15	2.9	2.0	13	*F*_(1,27)_ = 2.43, *p* = .13
Post	4.4	2.8	11	1.8	1.6	11	*F*_(1,22)_ = 6.26, *p* < .03
Hyperactivity (SDQ)							
Pre	6.3	2.8	15	5.4	3.1	13	*F*_(1,27)_ = .64, *p* = .43
Post	6.2	2.4	11	3.2	2.2	12	*F*_(1,22)_ = 18.28, *p* < .0004

Pre = pretreatment (i.e. at randomization); Post = post‐CT‐PTSD/WL (Week 11); 6MFU = 6‐month follow‐up; 12MFU = 12‐month follow‐up; CPSS, Child PTSD Symptom Scale; CT‐PTSD, Cognitive Therapy for PTSD; SDQ, Strengths and Difficulties Questionnaire; WL, waiting list.

a At pretreatment, one‐way ANOVA; at posttreatment, one‐way ANCOVA (pretreatment scores as covariates).

### Primary outcome: child‐report PTSD‐AA at posttreatment

The ITT analysis of child‐reported PTSD‐AA at posttreatment showed a significant between‐groups difference using both the WCS procedure [*χ* ^2^ = 5.81, *df* = 1, *p* < .02, 95% CI for difference .04–.71; assumes 10/14 (71%) diagnosis free for CT‐PTSD vs. 4/15 (27%) for WL] and the LOCF procedure [*χ* ^2^ = 10.08, *df* = 1, *p* < .002, 95% CI for difference .18–.80; assumes 10/14 (71%) diagnosis free for CT‐PTSD vs. 2/15 (13%) for WL] to account for dropouts.

Completer analyses also showed that CT‐PTSD participants were significantly more likely to be free of PTSD‐AA than WL [10/13 (77%) vs. 2/13 (15%) diagnosis free; *χ* ^2^ = 9.90, *df* = 1, *p* = .002; 95% CI for difference .18–.83] at posttreatment.

Similar results in favour of CT‐PTSD emerged using ICD‐10 PTSD as the outcome: ITT analysis using both WCS [*χ* ^2^ = 7.74, *df* = 1, *p* < .006, 95% CI for difference .10–.75; assumes 10/14 (71%) for CT‐PTSD vs. 3/15 (20%) for WL diagnosis free] and LOCF [*χ* ^2^ = 12.90, *df* = 1, *p* < .0001, 95% CI for difference .25–.85; assumes 10/14 (71%) vs. 1/15 (20%) diagnosis free], as well as completer‐only analysis [*χ* ^2^ = 12.76, *df* = 1, *p* < .0001, 95% CI for difference .32–.85; 10/13 (77%) vs. 1/13 (8%) diagnosis free].

### Secondary outcomes: PTSD symptoms and severity

For ITT analyses, the CT‐PTSD group scored significantly lower than the WL group on symptom counts (CPTSDI; mean difference 7.39, 95% CI 4.41–10.37; *p* < .0001) and PTSD severity (CPSS; mean difference 17.69, 95% CI 8.62–26.76; *p* < .0005). Results for completer analyses were consistent with ITT results (Table 2).

### Secondary outcomes: Clinically significant change analyses

Significantly more cases showed a clinically significant change for PTSD severity in the CT‐PTSD condition than WL [CPSS scores; 11/14 (78.6%) vs. 5/15 (33.3%); Fisher's exact test, *p* < .03].

### Secondary outcomes: other psychopathology and functioning

At posttreatment, the CT‐PTSD group had significantly better functioning (CGAS; mean difference 21.01, 95% CI 9.72–32.29; *p* < .0008), depression (MFQ; mean difference 14.63, 95% CI .59–28.67; *p* < .05), anxiety (SCAS; mean difference 19.15, 95% CI 5.72–32.57; *p* < .007), and parent‐reported emotional difficulties (SDQ; mean difference 2.77, 95% CI .60–4.93; *p* < .02), conduct problems (SDQ; mean difference 1.73, 95% CI .28–3.18; *p* < .03) and hyperactivity (SDQ; mean difference 3.27, 95% CI 1.58–4.94; *p* < .0005) relative to WL. Results for completer analyses were consistent with ITT results (Table 2).

### Treatment credibility and therapeutic alliance

Across all assessments CT‐PTSD participants condition rated their treatment as highly credible (i.e. first treatment session, midtreatment and posttreatment; range 36.1–38.3, possible range 4–40) and their therapeutic alliance as strong (TASC‐r scores: range 43.1–43.7, possible range 12–48).

### Six‐ and 12‐month follow‐up assessments

Of 13 CT‐PTSD cases re‐assessed at 6MFU and 12MFU, only one continued to meet criteria for PTSD‐AA; using the WCS procedure, 12/14 (86%) of all CT‐PTSD cases were diagnosis free at follow‐up. According to repeated‐measures ANOVAs for completers, this group also remained improved on continuous measures of PTSD, depression and anxiety at 6MFU and 12MFU assessments (*F* > 16.71, *p* < .002), compared to pretreatment (Table 2).

### Effect sizes

Between‐group (CT‐PTSD vs. WL at posttreatment) and within‐subjects (pre–post) effect sizes are presented in Table 3. Mean scores for ITT are presented in Table S1, available online. On an ITT basis, the CT‐PTSD group consistently showed large effect sizes for improvements relative to pretreatment scores and the WL group at posttreatment on all outcomes. For ITT analyses the WL group also experienced small to medium effect size improvements for PTSD symptomatology, but small or no improvement on other indices.

**Table 3:** Effect sizes on outcome measures for intent‐to‐treat and completer analyses

	Post‐treatment			Follow‐up	
	WL (pre to post)^a^	CT‐PTSD (pre to post)^a^	CT‐PTSD vs. WL (post)^b^	CT‐PTSD (pre to 6MFU)^a^	CT‐PTSD (pre to 12MFU)^a^
Intent‐to‐treat					
PTSD symptoms (CPTSDI)	.48	2.78	2.01	3.55	2.86
PTSD severity (CPSS)	.53	2.65	1.23	3.21	3.63
Depression (MFQ)	.11	1.43	.84	1.59	2.17
Anxiety (SCAS)	.25	1.40	1.56	1.70	2.09
Functioning (CGAS)	−.23	1.73	1.58		
Emotional problems (SDQ)	.07	1.20	1.02		
Conduct problems (SDQ)	−.07	.56	1.20		
Hyperactivity (SDQ)	−.09	.92	1.51		
Completer					
PTSD symptoms (CPTSDI)	.43	2.71	1.91	3.54	2.85
PTSD severity (CPSS)	.41	2.68	1.34	3.15	3.63
Depression (MFQ)	.14	1.41	.77	1.60	2.20
Anxiety (SCAS)	.15	1.43	1.09	1.74	2.12
Functioning (CGAS)	−.27	1.71	1.58		
Emotional problems (SDQ)	.21	1.11	.82		
Conduct problems (SDQ)	−.06	.61	1.12		
Hyperactivity (SDQ)	.03	.77	1.26		

Pre = pretreatment (i.e. at randomization); Post = post‐CT‐PTSD/WL (Week 11); 6MFU = 6‐month follow‐up; 12MFU = 12‐month follow‐up; CPSS, Child PTSD Symptom Scale; CT‐PTSD, Cognitive Therapy for PTSD; SDQ, Strengths and Difficulties Questionnaire; WL, waiting list.

a Within‐group; negative scores indicate worsening symptomatology/functioning.

b Between‐group; positive scores indicate superiority of CT‐PTSD.

### Mediation analysis

Pre‐, mid‐ and posttreatment data on putative mediator variables, with between‐group comparisons, are displayed in Table S2; the WL group scored significantly higher than the CT‐PTSD group on each variable at posttreatment (all *p* < .03). Correlations between pre–post changes in CPSS scores and pre–post changes in potential mediators were large and significant for trauma‐related appraisals, memory quality, trauma‐related rumination and safety‐seeking behaviours [*rs*(26) = .48–67, *p* < .02], but not self‐blame or social support (MSPSS; *r* = −.10 for each variable, *p* > .63). These latter variables were, therefore, not considered further.

Our first mediation strategy addressed whether the relationship between allocation and pre–post change in PTSD symptomatology was mediated by pre–post change in our putative mediators (see Figure S1a for pathways, Table S3 for coefficients). Pre–post changes in trauma‐related misappraisals, memory quality, rumination and safety‐seeking behaviours were significant mediators of the relationship between treatment allocation and pre–post change in CPSS scores.

The second mediation strategy considered whether *pre–mid* changes in our putative mediator variables mediated the relationship between allocation and CPSS scores at posttreatment (see Figure S1b for pathways and Table S3 for coefficients). According to this method, trauma‐related misappraisals and safety‐seeking behaviours, but not memory quality or rumination, were significant mediators.

## Discussion

Consistent with our hypotheses, this RCT provided preliminary support for the efficacy of CT‐PTSD as a treatment for youth with PTSD in the first 2‐ to 6‐months post‐trauma. Relative to a WL, at posttreatment CT‐PTSD led to greater loss of an age‐appropriate PTSD diagnosis (and of an ICD‐10 diagnosis) and reduced PTSD symptoms, as well as significant improvements in depression, anxiety, clinician‐rated functioning and other comorbidity. There was also good preliminary support for the role of psychological mechanisms in mediating the relationship between allocation and outcome, consistent with the stated targets of CT‐PTSD (Smith et al., 2010). This is the first study to show the efficacy of a psychological treatment over natural recovery for the early treatment of PTSD in youth and the first study to show the efficacy of any treatment for PTSD in youth in the early post‐trauma window of 2–6 months.

Effect sizes for CT‐PTSD in this trial are comparable to the earlier evaluations of CT‐PTSD for youth with chronic PTSD (Smith et al., 2007), CT‐PTSD as an early treatment for adult PTSD (Ehlers et al., 2003) and trauma‐focused cognitive‐behavioural therapy for multiple‐trauma PTSD such as child sexual abuse (Cohen et al., 2004). CT‐PTSD was also very acceptable to trial participants, with only one drop out and high ratings for treatment credibility and therapist alliance. In summary, these data suggest CT‐PTSD is a potentially powerful and acceptable early treatment for youth with PTSD that may yield sustained improvements and benefit broader mental health and functioning.

Youth in the WL arm experienced some nontrivial recovery (33% experienced clinically significant change), at a level broadly comparable with other WL‐controlled RCTs (Goldbeck, Muche, Sachser, Tutus, & Rosner, 2016; King et al., 2000; Smith et al., 2007). Nevertheless, this RCT suggests that youth identified as having PTSD in the 2‐ to 6‐months window post‐trauma are more likely than not to require treatment. This was particularly apparent when considering functioning and depression data, which showed considerable improvement in the CT‐PTSD arm but no change in WL. Given the scale of the issue and the risk of long‐term poor functioning, clearly more research is needed concerning how treatment in this window might be delivered more cost‐effectively.

This study provides support for the role of cognitive mechanisms in treatment responsiveness. The mediation effect for misappraisals replicated a previous finding with chronic PTSD youth (Smith et al., 2007). This study adds to previous findings by preliminarily demonstrating the effect of these mechanisms relative to other plausible but nontheory‐derived mechanisms, extending the range of cognitive mechanisms considered (memory characteristics, rumination and safety‐seeking behaviours) and utilizing a more robust mediation analysis procedure. When using this procedure, only misappraisals and safety‐seeking behaviours showed a mediation effect. These data provide additional support for the utility of a cognitive model of PTSD in youth and suggest further research into these mechanisms is warranted; such mechanisms may be possible treatment targets for more easily disseminated treatments (e.g. computerized or self‐help therapy packages).

This trial has several limitations. While in line with our prestudy power calculation and adequately powered to reveal significant treatment effects of CT‐PTSD, the limited sample size rendered the study susceptible to bias (e.g. the unbalanced ethnicity, a trend towards unbalanced socioeconomic characteristics). It is unclear whether the limited representation of males in the trial sample (only 27%) also reflects this issue, or results from problems engaging with this group. The generalizability of the study findings is limited by the use of well‐supervised clinical psychologists who were very familiar with a cognitive understanding of PTSD in youth. Some of the measures used to assess mediation effects did not have good internal consistency in this sample. While the WL arm may reflect a ‘watchful waiting’ approach, it is unclear how well the WL arm reflects ‘natural recovery’ (as participants knew they would receive treatment soon). Treatment fidelity was not formally assessed using a sessional coding system.

Future evaluations of CT‐PTSD, for acute or chronic PTSD, now need to consider its utility relative to an ‘active’ control condition including therapist contact (e.g. supportive counselling), and consider its role as part of stepped‐care and indicated interventions for trauma‐exposed youth that may be more cost‐effective. Careful attention to recruitment and engagement issues will be required for any future evaluation of CT‐PTSD as an early treatment. Despite recruiting from a wide area (the counties of Cambridgeshire, Norfolk, Suffolk, Essex, Bedfordshire and Hertfordshire in England, comprising several hundred thousand youth in the target age range) and having 29 months to recruit, recruitment rate was slow. While research into potential barriers to seeking or accessing care is needed (e.g. parental understanding, more widespread use of screening tools, referral pathways), these data may alert clinicians and referrers to the possibility of successful early treatment for PTSD that results from commonly occurring traumatic stressors.

**Key points**

- Early interventions for trauma‐exposed youth to date have focused on the prevention of PTSD in the first few weeks following a trauma with limited success.
- A multiple‐session individual psychological treatment, Cognitive Therapy for PTSD (CT‐PTSD), delivered in the first 2‐ to 6‐month post‐trauma, was efficacious relative to a wait list (WL) control arm.
- Recovery in the WL arm was limited (particularly in terms of depression and functioning), suggesting that further monitoring in this period is not warranted.
- Mediation analysis suggested that CT‐PTSD works through reducing trauma‐related misappraisals and safety‐seeking behaviours.

## Supporting information


**Figure S1.** Putative mediation pathways.

**Table S1.** Scores on outcome measures at posttreatment and follow‐up based on multiple imputation.
**Table S2.** Mediator measures for completers at each assessment.
**Table S3.** Mediators of treatment responsiveness.

**Appendix S1.** CONSORT 2010 checklist of information to include when reporting a randomized trial.
