# Supplementary material for: Cognitive therapy as an early treatment for post‐traumatic stress disorder in children and adolescents: a randomized controlled trial addressing preliminary efficacy and mechanisms of action
Source: J Child Psychol Psychiatry. 2016 Dec 15;58(5):623–33. doi: 10.1111/jcpp.12673 (PMC5362068; doi:10.1111/jcpp.12673)
Supplement: Supplementary file 1 — Figure S1. Putative mediation pathways. [file JCPP-58-623-s001.docx]

**Figure S1. Putative mediation pathways**

1. Pre-post change in putative mediator variables as mediator between allocation and pre-post change in PTSD severity

IV: Allocation (CT-PTSD vs WL)

DV: Pre-post change in PTSD severity

c' path (direct effect)

b path

M: Pre-post change in putative mediator

a path

c path (total effect)

DV: Pre-post change in PTSD severity

IV: Allocation (CT-PTSD vs WL)

1. Pre-mid change in putative mediator variables as mediator between allocation and PTSD severity at post-treatment

M: Pre-mid change in putative mediator

b path

IV: Allocation

(CT-PTSD vs WL)

c' path (direct effect)

DV: PTSD severity at post-treatment

DV: PTSD severity at post-treatment

a path

c path (total effect)

IV: Allocation (CT-PTSD vs WL)

Note. IV = Independent Variable; M = Mediator; DV = dependent variable.
