# Supplementary material for: Cognitive therapy as an early treatment for post‐traumatic stress disorder in children and adolescents: a randomized controlled trial addressing preliminary efficacy and mechanisms of action
Source: J Child Psychol Psychiatry. 2016 Dec 15;58(5):623–33. doi: 10.1111/jcpp.12673 (PMC5362068; doi:10.1111/jcpp.12673)
Supplement: Supplementary file 2 — Table S1. Scores on outcome measures at posttreatment and follow‐up based on multiple imputation. Table S2. Mediator measures for completers at each assessment. Table S3. Mediators of treatment responsiveness. [file JCPP-58-623-s002.docx]

**Supporting information for *Cognitive Therapy as an early treatment for post-traumatic stress disorder in children and adolescents: a randomized controlled trial addressing preliminary efficacy and mechanisms of action* by Meiser-Stedman et al.**

Table S1. Scores on outcome measures at post-treatment and follow up based on multiple imputation

|  | WL | | | CT-PTSD | | |  |
| --- | --- | --- | --- | --- | --- | --- | --- |
| Outcome variable | M | SD | N | M | SD | N | Group effect ^a^ |
| PTSD symptoms (CPTSDI) |  |  |  |  |  |  |  |
| Post | 10.1 | 4.0 | 15 | 2.6 | 3.4 | 14 | F_1,26_ = 26.05, p<.0001 |
| 6MFU | - | - | - | 1.6 | 2.5 | 14 | - |
| 12MFU | - | - | - | 2.2 | 3.5 | 14 | - |
| PTSD severity (CPSS) |  |  |  |  |  |  |  |
| Post | 22.9 | 15.4 | 15 | 6.8 | 10.3 | 14 | F_1,26_ = 17.69, p<.0005 |
| 6MFU | - | - | - | 5.3 | 8.2 | 14 | - |
| 12MFU | - | - | - | 5.8 | 6.0 | 14 | - |
| Depression (MFQ) |  |  |  |  |  |  |  |
| Post | 25.7 | 20.1 | 15 | 11.3 | 13.5 | 14 | F_1,26_ = 4.59, p<.05 |
| 6MFU | - | - | - | 11.7 | 10.6 | 14 | - |
| 12MFU | - | - | - | 8.5 | 7.0 | 14 | - |
| Anxiety (SCAS) |  |  |  |  |  |  |  |
| Post | 48.4 | 32.1 | 15 | 20.4 | 17.6 | 14 | F_1,26_ = 8.59, p<.007 |
| 6MFU | - | - | - | 17.0 | 15.1 | 14 | - |
| 12MFU | - | - | - | 14.8 | 9.9 | 14 | - |
| Functioning (CGAS) ^b^ | 55.7 | 13.4 | 15 | 77.1 | 13.7 | 14 | F_1,26_ = 14.66, p<.0008 |
| Parent-report emotional difficulties (SDQ) ^b^ | 6.1 | 3.3 | 15 | 3.3 | 2.0 | 14 | F_1,26_ = 6.91, p<.02 |
| Parent-report conduct problems (SDQ) ^b^ | 4.4 | 2.4 | 15 | 1.9 | 1.6 | 14 | F_1,26_ = 6.05, p<.03 |
| Parent-report hyperactivity (SDQ) ^b^ | 6.5 | 2.2 | 15 | 3.1 | 2.2 | 14 | F_1,26_ = 16.01, p<.0005 |

Note. WL = wait list; CT-PTSD = Cognitive Therapy for PTSD; Post = post-CT-PTSD/WL (week 11); 6MFU = six month follow-up; 12MFU = 12 month follow up; CPTSDI = Children’s PTSD Inventory; CPSS = Child PTSD Symptom Scale; MFQ = Mood and Feelings Questionnaire; SCAS = Spence Child Anxiety Scale; CGAS = Clinician’s Global Assessment Scale; SDQ = Strengths and Difficulties Questionnaire.

^a^ All analyses are ANCOVA, with pre-treatment scores as covariates. ^b^ Post-treatment only.

Table S2. Mediator measures for completers at each assessment

|  | WL | | | CT-PTSD | | |  |
| --- | --- | --- | --- | --- | --- | --- | --- |
| Outcome variable | M | SD | N | M | SD | N | Group effect ^a^ |
| Misappraisals (CPTCI) |  |  |  |  |  |  |  |
| Pre | 64.6 | 22.8 | 15 | 60.5 | 15.8 | 14 | F_(1,28)_ = .30, p=.59 |
| Mid | 60.6 | 27.7 | 15 | 46.1 | 19.6 | 13 | F_(1,27)_ = 2.09, p=.16 |
| Post | 62.9 | 27.6 | 13 | 38.9 | 15.5 | 13 | F_(1,25)_ = 7.57, p<.02 |
| Memory quality (TMQQ) |  |  |  |  |  |  |  |
| Pre | 32.8 | 4.6 | 15 | 33.7 | 4.3 | 14 | F_(1,28)_ = .30, p=.59 |
| Mid | 28.5 | 8.7 | 15 | 27.9 | 7.1 | 13 | F_(1,27)_ = .15, p=.70 |
| Post | 30.8 | 7.9 | 13 | 21.7 | 4.9 | 13 | F_(1,25)_ = 14.29, p<.001 |
| Rumination |  |  |  |  |  |  |  |
| Pre | 9.5 | 2.5 | 15 | 10.2 | 1.6 | 14 | F_(1,28)_ = .90, p=.35 |
| Mid | 10.0 | 2.4 | 15 | 8.8 | 2.7 | 13 | F_(1,27)_ = 1.95, p=.18 |
| Post | 10.0 | 1.6 | 13 | 6.7 | 2.7 | 13 | F_(1,25)_ = 21.39, p<.0001 |
| Safety-seeking behavs |  |  |  |  |  |  |  |
| Pre | 36.3 | 17.2 | 15 | 34.3 | 14.2 | 14 | F_(1,28)_ = .12, p=.74 |
| Mid | 36.0 | 21.5 | 15 | 23.1 | 13.7 | 13 | F_(1,27)_ = 3.47, p=.07 |
| Post | 35.2 | 20.3 | 13 | 18.5 | 15.1 | 13 | F_(1,25)_ = 5.68, p<.03 |
| Self-blame |  |  |  |  |  |  |  |
| Pre | 4.2 | 2.5 | 15 | 3.1 | 1.6 | 14 | F_(1,28)_ = 1.88, p=.18 |
| Mid | 3.9 | 2.4 | 15 | 2.6 | 1.2 | 13 | F_(1,27)_ = 1.51, p=.23 |
| Post | 4.0 | 2.7 | 13 | 2.8 | 1.5 | 13 | F_(1,25)_ = .29, p=.60 |
| Social support (MSPSS) |  |  |  |  |  |  |  |
| Pre | 62.9 | 19.5 | 15 | 65.0 | 13.2 | 14 | F_(1,28)_ = .12, p=.73 |
| Post | 69.5 | 12.6 | 13 | 67.5 | 16.1 | 13 | F_(1,25)_ = .32, p=.58 |

Note. ^a^ At pre-treatment, one-way analysis of variance; at mid- and post-treatment, one-way analysis of covariance with pre-treatment scores as covariates. WL = wait list; CT-PTSD = Cognitive Therapy for PTSD; Pre = pre-treatment (i.e. at randomisation); Mid = five weeks post-randomisation; Post = post-CT-PTSD/WL (week 11); CPTCI = Child Post-Traumatic Cognitions Inventory; TMQQ = Trauma Memory Quality Questionnaire; MSPSS = Multidimensional Scale of Perceived Social Support.

Table S3. Mediators of treatment responsiveness

| Mediator | a path | b path | c path | c' path | Indirect effect  (c – c') | 95% CI of indirect effect | R^2^ |
| --- | --- | --- | --- | --- | --- | --- | --- |
|  | | | | | | | |
| *Analysis 1: Mediators of relationship between trial allocation and pre-post change in PTSD severity (CPSS) – the role of pre-post changes in cognitive mechanisms* | | | | | | | |
| Misappraisals |  |  |  |  |  |  |  |
| Δ Misappraisals (CPTCI) | 20.18^**^ | .40^**^ | 16.75^***^ | 8.67^*^ | 8.09 | (2.57, 17.02) | .66 |
| Memory quality |  |  |  |  |  |  |  |
| Δ Memory Quality (TMQQ) | 8.68^***^ | 1.31^***^ | 17.20^***^ | 5.86 | 11.34 | (5.16, 29.51) | .66 |
| Dysfunctional coping |  |  |  |  |  |  |  |
| Δ Rumination | 3.46^***^ | 2.61^*^ | 17.30^***^ | 8.26 | 9.04 | (2.97, 18.29) | .54 |
| Δ Safety-seeking behav. | 12.58^*^ | .52^**^ | 15.06^***^ | 8.55 | 6.51 | (2.04, 15.00) | .74 |
|  |  |  |  |  |  |  |  |
| *Analysis 2: Mediators of relationship between allocation and PTSD severity (CPSS) at post-treatment – the role of pre-mid changes in cognitive mechanisms.* | | | | | | | |
| Misappraisals |  |  |  |  |  |  |  |
| Δ Misappraisals (CPTCI) | 12.20 | -.42^***^ | -16.75^***^ | -11.57^**^ | -5.18 | (-12.63, -.25) | .80 |
| Memory quality |  |  |  |  |  |  |  |
| Δ Memory Quality (TMQQ) | .06 | -.35 | -17.20^***^ | -17.18^***^ | -.02 | (-2.82, 4.01) | .62 |
| Dysfunctional coping |  |  |  |  |  |  |  |
| Δ Rumination | 1.27 | -2.42^**^ | -17.30^***^ | -14.23^***^ | -3.07 | (-10.21, .27) | .74 |
| Δ Safety-seeking behav. | 9.78 | -.37^*^ | -15.06^***^ | -11.46^**^ | -3.60 | (-13.00, -.10) | .77 |

Note. a path = independent variable (IV; allocation) to mediator; b path = direct effect of mediator on dependent variable (DV; pre-post change in CPSS or CPSS at post-treatment); c path = total effect of IV on DV; c' path = direct effect of IV on DV; CPTCI = Child Post-Traumatic Cognitions Inventory; TMQQ = Trauma Memory Quality Questionnaire. Pre-treatment symptom counts and corresponding mediator scores were entered as covariates into each model. Mediation is indicated where the 95% confidence intervals of the indirect effect do not cross zero.

^*^ = p<.05; ^**^ = p<.01; ^***^ = p<.001.
